# The prevalence of risk factors associated with non-communicable diseases in Afghan refugees in southern Iran: a cross-sectional study

**DOI:** 10.1186/s12889-021-10482-9

**Published:** 2021-03-05

**Authors:** Erfan Taherifard, Mohammad Javad Moradian, Ehsan Taherifard, Abdolrasool Hemmati, Behnaz Rastegarfar, Hossein Molavi Vardanjani

**Affiliations:** 1 MPH Department, Medical School, Shiraz University of Medical Sciences, Setad Square, Shiraz, Fars Iran; 2 Trauma Research Center, Shahid Rajaee (Emtiaz) Trauma Hospital, Shiraz University of Medical Sciences, Shiraz, Iran; 3 Shiraz University of Medical Sciences, Shiraz, Iran; 4 Department of Disaster Public Health, School of Public Health, Tehran University of Medical Sciences, Tehran, Iran

**Keywords:** Health survey, Refugees, Risk factor, Non-communicable diseases

## Abstract

**Background:**

Refugees are highly vulnerable to many health-related risks. Monitoring non-communicable diseases (NCDs) is of overriding importance in these populations. This study aimed to investigate the prevalence of risk factors for NCDs amongst Afghan refugees in a refugee camp located in southern Iran.

**Methods:**

This cross-sectional sturdy was conducted in 2018. Risk factors such as inadequate nutrition, physical inactivity, tobacco smoking, obesity and overweight, hypertension (HTN), elevated fasting plasma glucose (FPG), and dyslipidaemia were assessed. Data were gathered with a modified WHO STEPS procedure. Prevalence and age-standardized prevalence and their 95% confidence intervals (CI) were estimated.

**Results:**

The estimated prevalence were 94% for inadequate fruit/vegetable consumption, 18% for physical inactivity, 9% for tobacco smoking, 3% for FPG, 20% for HTN, 51% for central obesity, 24% for overweight, 19% for obesity, and 69% for dyslipidaemia.

**Conclusions:**

Except for inadequate fruit and vegetable intake and dyslipidaemia, the prevalence of other NCD risk factors was low among Afghan refugees in Iran. Raising awareness about healthy diet and its importance and the provision of more affordable fruit and vegetables are two effective measures toward improving the health of refugees in Iran.

## Background

Refugees, as a vulnerable population [1], have higher risks of developing mental [2, 3] and physical [4] disorders [5]. In addition to higher rates of infectious diseases [6], the prevalence of non-communicable diseases (NCDs) and their risk factors are rising in these populations [7, 8].

The largest and fastest growing proportion of the global burden of diseases is attributed to NCDs [9, 10], particularly in low- and middle-income populations. Therefore NCD control is a worldwide priority [11]. Controlling NCDs requires a shift to primary prevention by tackling key modifiable risk factors including inadequate nutrition, physical inactivity, alcohol consumption, smoking, high blood pressure, and dyslipidaemia [12, 13]. Therefore, monitoring the current status of these key risk factors is an essential element of NCD control.

The Islamic Republic of Iran has been hosting Afghan refugees since the 1980s [14]. In July 2017, it was reported by the United Nations High Commissioner for Refugees (UNHCR) that more than 950,000 Afghan refugees were residing Iran [15]. Most of this population consists of the progeny of two main waves of asylum seekers who fled Afghanistan and entered Iran after the invasion of Soviet troops in 1979 and during the Taliban rule era from 1996 to 2001 [16, 17]. However, the majority of them are unable to gain citizenship [18], and so are not included in health screening programs such as STEPwise approach to surveillance (STEPS). Accordingly, the current status and prevalence of key risk factors of NCDs are unknown among Afghan refugees in Iran.

To date, seven rounds of national surveillance studies of risk factors for NCDs have been conducted in Iran since 2005. According to the latest study in 2016, inadequate fruit and vegetable intake, abnormal lipid profile and insufficient physical activity comprised the three most prevalent risk factors among the Iranian population at 82, 69 and 56%, respectively [19]. Although there is no national NCDs surveillance in Afghanistan, two studies assessed the prevalence of risk factors for NCDs in Jalalabad and Kabul [20, 21]. In the Kabul study it was reported that the prevalence of obesity and hypertension (HTN) were 31 and 33%, respectively.

The present study was conducted to assess the current status of prevalence of the most common risk factors for NCDs with a modified World Health Organisation (WHO) STEPS procedure.

## Methods

This survey was conducted in one of the eight registered Afghan refugee camps, located in southern Iran. This camp was selected as a convenient study setting. Because of executive and legal issues the study had not applicability to be conducted in other camps. In this camp, almost all primary and outpatient healthcare services are provided by a single healthcare centre funded by the UNHCR. One general practitioner and four healthcare providers, including two Afghans residing in the camp, are responsible for providing healthcare for this population. A total of 628 Afghan refugees reside in the camp (327 men and 301 women); almost all female individuals in this population do not work outside their house, and the men are the breadwinners of the family, engaging mostly in manual labour occupations.

### Sample size and participants

All camp residents who were 15 years of age or older were eligible to participate in the study, and all were enrolled.

### Data collection

The study was designed mainly based on the WHO STEPwise approach to NCD risk factors surveillance [22]. The data were collected by the camp’s healthcare providers in 2018. A 2-day training workshop was held for the staff before data collection, and their first day of data collection was supervised by 2 Ministry of Health experts.

Data were collected in three steps as follows: step 1: a gender-matched face-to-face interview for demographic information and behavioural measurements; step 2: physical and anthropometric measurements; step 3: biochemical measurements. All participants were invited to come to the clinic early in the morning, and were instructed to fast for at least 10 h in preparation for step 3. Furthermore, individuals with diabetes who were on medication (tablets or insulin) were reminded not to forget their medications and to bring them to the laboratory for subsequent use after step 3 was completed.

#### Step 1: demographic information and behavioural measurements

During an interview based on the Iranian national STEPS interview form, participants were asked about the number of members in the household, house residential area, average household monthly income, and their gender and age. Data on their nutrition, physical activity, and tobacco consumption were also collected. For nutritional status, the numbers of fruits and vegetables consumed on average in a typical day of the week were recorded. For physical activity, the participants were questioned about the quality and quantity of physical activity in the three domains of occupation, transport (walking, hiking or cycling) and recreation. Occupational physical activities were divided into two categories of vigorous or moderate intensity. Vigorous-intensity activities were those which lasted for at least 10 min continuously and caused a subjective perception of marked increase in respiration and heart rate (for example, carrying heavy loads, construction, forestry and drilling). Moderate-intensity activities lasted for at least 10 min and led to a subjective perception of slight increase in breathing and heart rate (for example, cleaning, washing by hand, milking cows by hand, planting and harvesting crops, and weaving). In a same way, activity during leisure time was recorded as two types: vigorous-intensity activities which made participants breathe much harder than normal, such as soccer, tennis, high-impact aerobics, and fast swimming, or moderate-intensity ones which made participants breathe somewhat harder than normal, such as cycling, jogging, and low-impact aerobics.

Furthermore, respondents were asked if a medical practitioner or healthcare provider had ever told them that they had diabetes mellitus (DM) or HTN.

#### Step 2: physical measurements

Height and weight were measured with a mechanical telescopic measuring rod (Seca 222, Germany) and a digital scale (EF972, China), respectively. For height measurements, the participants were asked to remove their footwear and head gear, and those with a high hairdo were asked to press it down. In women, height was measured by female staff members in order to avoid awkwardness if participants needed to remove their veil or head scarf. To record body weight, all participants were asked the day before to wear light clothes, and were asked to remove their shoes and head gear, and to empty out their pockets.

For waist (WC) and hip circumference (HP) measurements, a constant tension tape was placed on the midpoint between the lowest palpable rib and the highest part of the iliac crest, and participants were asked to relax their arms at their sides; then WC was measured at the end of a normal expiration. Then the tape was positioned at the widest part of the buttocks, and HP was measured while their arms were relaxed at their sides.

Blood pressure (BP) was measured twice for each participant, once after step 1 was completed, and again at the end of step 3. An automatic digital sphygmomanometer (ALPK2 K2 1702) was used for BP measurements.

#### Step 3: biochemical measurements

A 5-mL sample of venous blood was collected in a tube while the participant was seated, and serum was separated by centrifugation immediately thereafter. Fasting plasma glucose (FPG), triglycerides (TG), total cholesterol (TC), high-density lipoprotein cholesterol (HDL-C), and low-density lipoprotein cholesterol (LDL-C) were measured with a chemical auto-analyser (Tokyo Boeki Prestige 24i, Japan). To determine serum levels of FPG and lipid profile, an enzymatic colorimetric method and enzymatic method respectively were used.

### Definition of variables

All definitions were adopted from standard WHO definitions [23–25] as detailed below:

1. Unhealthy diet or inadequate fruit/vegetable consumption was defined as an average intake of fruit and vegetables of fewer than 5 servings per day [24].
2. Insufficient physical activity was considered as less than 75 min of vigorous-intensity physical activity or less than 150 min of moderate-intensity activity, or an equivalent combination of vigorous- and moderate-intensity physical activity to yield 600 MET-minutes during a typical week.
3. Elevated plasma glucose was defined as a plasma level ≥ 7.0 mmol/L (126 mg/dL), and was also recorded if the participant was currently on medication for DM. The prediabetes stage was defined as a plasma glucose level between 5.5 and 7.0 mmol/L (100–125 mg/dL) [24, 26].
4. Elevated blood pressure was defined as systolic blood pressure (SBP) ≥140 mmHg and/or diastolic blood pressure (DBP) ≥90 mmHg. Participants who were on medication for HTN were also considered to have elevated blood pressure. A SBP between 120 and 139 mmHg or a DBP between 80 and 89 mmHg were recorded as prehypertension stage [24].
5. Individuals who met at least 3 of the following criteria were recorded as having metabolic syndrome: WC more than 89 cm in men and more than 79 cm in women, elevated TG (≥150 mg/dL), low HDL (< 40 mg/dL in men and < 50 mg/dL in women), SBP ≥ 130 mmHg and/or DBP ≥ 85 mmHg, or current use of medication for HTN, plasma glucose > 5.5 mmol/L, or current medication for DM [27].
6. Abdominal obesity (abnormal waist-to-hip ratio) was defined as a waist-hip ratio > 0.90 in males and > 0.85 in females [27].
7. Abnormal waist-to-height ratio was considered waist-to-height ratio of more than 0.55 in men and more than 0.62 in women [28].
8. Body mass index was divided into four categories: below 18 kg/m^2^, between 18 kg/m^2^ and 25 kg/m^2^, between 25 kg/m^2^ and 30 kg/m^2^, and above 30 kg/m^2^. These categories were considered respectively as underweight, healthy weight, overweight, and obese [27].
9. Abnormal lipid profile was defined as any of the followings: elevated TC (≥ 200 mg/dL), high LDL (≥ 160 mg/dL), low HDL (< 40 mg/dL in men and < 50 mg/dL in women), or elevated TG (≥ 150 mg/dL) [29].

### Data cleaning and statistical analysis

The data were cleaned with appropriate techniques as described previously [24]. Descriptive statistics were estimated. The world standard population distribution in 2000–25 was used to estimate the age-standardized prevalence of NCD risk factors and their confidence intervals. Crude and age-standardized prevalence and their Poisson 95% confidence intervals (95% CI) were estimated for abnormal levels of each risk factor. The chi-squared test was used to evaluate the association of behavioural, physical and biological variables with age and gender. A *p*-value less than 0.05 was considered statistically significant. Statistical procedures were applied with Stata software (StataCorp SE, College Station, TX, USA).

### Ethical consideration

Because the refugees in our study population were familiar with the clinical staff, the purpose and procedure of the study were explained to participants by the staff, and then written informed consent was obtained from all participants. In addition, within the camp each house had a unique code. We agreed that because of ethical considerations, the healthcare providers would use this code on the data sheets as an identifier to enhance anonymity, so that the researchers did not have access to the participants’ names. The study was approved by the ethics committee of Shiraz University of Medical Sciences.

## Results

The study population comprised all refugees older than 15 years in 133 families, and each family lived in a different house. There were 114 men with a mean age of 37.4 (±17.3) years, and 162 women with a mean age of 36.1 (±13.93) years. They were divided into 4 age groups: 15–24, 25–44, 45–64, and above 65 years.

The overall prevalence of consuming fewer than 5 servings of fruit/vegetables daily was 94.5% (94.7% in men and 94.4% in women; the difference between these two groups was not statistically significant at *p* = 0.916). In all age groups, the percentage of individuals with inadequate fruit/vegetable consumption was above 90%, and was highest (100%) among those more than 65 years old. As noted above for gender, there was no significant association between age and unhealthy diet (*p* = 0.582; Table 1).

**Table 1:** Behavioural, physical and biochemical risk factors for non-communicable diseases by age and gender group

Factor	No.	% Crude prevalence (CI)	% Standardized prevalence (CI)	Prevalence in age subgroups (%)							
				Men % (CI)	Women % (CI)	*P*-value	15–24	25–44	45–65	Above 65	*p*-value
Total number	276	100	100	114	162	–	69	132	57	18	–
Behavioural variables											
Fruit/vegetables < 5 servings	261	94 (91–96)	69 (67–71)	94 (88–98)	94 (89–97)	0.916	94	93	96	100	0.582
Insufficient physical activity	50	18 (13–23)	14 (10–18)	11 (6–18)	22 (16–30)	0.015	15	16	19	33	0.352
Tobacco use	26	9 (6–13)	8 (4–11)	18 (11–26)	3 (1–7)	< 0.001	10	9	10	5	0.928
Known diabetes	7	2 (1–5)	1 (0.3–3)	0.8 (0.02–4)	3 (1–7)	0.141	0	0.7	10	0	< 0.001
Prediabetes	16	5 (3–9)	4 (2–7)	4 (1–9)	6 (3–11)	0.400	5	3	10	5	0.346
Elevated fasting plasma glucose	9	3 (1–6)	2 (0.6–3)	1 (0.2–6)	4 (1–8)	0.237	1	1	10	0	0.007
Known HTN	45	16 (12–21)	13 (9–16)	14 (8–21)	17 (12–24)	0.392	1	9	38	55	< 0.001
Prehypertension	62	22 (17–27)	15 (11–19)	24 (16–33)	20 (14–28)	0.484	18	26	22	5	0.193
Elevated blood pressure	56	20 (15–25)	16 (12–19)	20 (13–28)	20 (14–27)	0.968	7	12	42	55	< 0.001
Metabolic syndrome	39	14 (10–18)	10 (7–13)	7 (3–13)	19 (13–26)	0.004	7	12	24	22	0.027
Physical variables											
Abnormal waist-to-hip ratio	141	51 (45–57)	37 (32–42)	39 (30–49)	59 (51–66)	0.001	28	51	77	50	< 0.001
Abnormal waist-to-height ratio	79	28 (22–35)	27 (22–32)	42 (31–55)	19 (13–27)	< 0.001	26	29	29	27	0.956
Metabolically healthy obesity	35	12 (8–17)	8 (5–11)	12 (6–19)	12 (8–19)	0.867	8	18	7	0	0.019
Underweight	16	5 (3–9)	6 (4–9)	7 (3–13)	4 (2–9)	0.467	11	1	5	16	0.006
Overweight	68	24 (19–30)	19 (15–23)	24 (16–33)	24 (18–32)	0.980	13	27	29	33	0.070
Obese	55	19 (15–25)	13 (10–16)	15 (9–23)	22 (16–30)	0.149	13	25	21	5	0.088
Biochemical variables											
Elevated total cholesterol	107	38 (32–44)	28 (23–33)	38 (29–44)	38 (31–46)	0.961	20	38	59	44	< 0.001
Elevated TG	36	13 (9–17)	9 (5–12)	11 (6–18)	14 (9–20)	0.497	2	18	12	16	0.023
High LDL	9	3 (1–6)	2 (0.6–3)	2 (0.5–7)	3 (1–7)	0.621	0	4	3	5	0.344
Low HDL	113	40 (35–46)	29 (25–34)	16 (10–24)	58 (50–65)	< 0.001	42	40	42	33	0.920
Abnormal lipid profile	191	69 (63–74)	49 (45–54)	54 (44–63)	79 (72–85)	< 0.001	55	68	84	77	0.004

The overall prevalence of insufficient physical activity was 18.1%; this prevalence was higher in women (22.8%, 37 individuals) than men (11.4%, 13 individuals), and the difference between these two groups was statistically significant at *p* = 0.015. Furthermore, the highest rate of physical inactivity was found among those older than 65 years (33.3%); however, no significant relationship was detected between age groups and low levels of physical activity (*p* = 0.352; Table 1).

The overall prevalence of tobacco consumption (both smoked and smokeless tobacco products) was 9.4%, with a significantly higher proportion in males (18.4%, 21 individuals) than females (3%, 5 individuals; *p* < 0.001). There were no statistically significant differences between age groups in the percentage of individuals who used tobacco products (*p* = 0.928; Table 1).

Elevated FPG was recorded in 2.8% (8 individuals) of the population, of whom 1.7% (2 individuals) were males and 3.7% (6 individuals) were females. Among these participants, 6 individuals were aware of their diabetes and were using medication. The prevalence of participants with elevated FPG was highest in the 45–64-year-old age group (*p* = 0.007; Table 1).

In the target population, 20.2% of the participants had elevated BP, with no statistically significant difference between men and women (*p* = 0.968). Eleven participants were unaware of having elevated BP. The percentage of participants with elevated BP showed a statistically significant relationship with age, and the prevalence was highest among individuals older than 65 years (*p* < 0.001; Table 1).

The prevalence of central obesity was 51.2%, and this prevalence was significantly higher in women than men (*p* = 0.001). There was also a statistically significant relationship between age group and the prevalence of central obesity (*p* < 0.001; Table 1).

In the target population, a total of 191 persons (69.2%) had at least one abnormality in lipid profile components (high TG, TC and LDL-C, and low HDL-C). The prevalence of abnormal profiles was statistically significantly higher in women than in men (p < 0.001). Similarly, age had a statistically significant relationship with lipid profile status (*p* = 0.004; Table 1).

Among behavioural, physical and biochemical risk factors, unhealthy diet, abnormal waist-to-hip ratio and abnormal lipid profile had the highest prevalence (Table 1).

## Discussion

The study showed that 94% of the Afghan refugees consumed fewer than 5 servings of fruit/vegetables on a typical day; this proportion reached 100% in individuals older than 65 years, among whom none reported that their diet met the recommendation proposed by the WHO. This prevalence of unhealthy diet composition was extremely higher than the prevalence of inadequate fruit/vegetable consumption in Iran [19]. One of the most important barriers is the lack of knowledge about recommended daily intakes; almost all the refugee population in the present study was unaware of such recommendations. The affordability of fruit and vegetables is another determinant of intake, and its influence has been exacerbated due to inflation and currency collapse as a result of economic sanctions.

Prevalence of low level of physical activity in the study population was only 18%. This is very lower than reported prevalence in Iran (56.17%) and also in Fars province (nearly 57%) [19]. One factor contributing to this discrepancy between the general population and refugee population is that unlike local residents, refugees are more often forced by necessity to accept hard and physically active jobs, such as carrying heavy loads, drilling and mining. Furthermore, the refugee population has far less access to facilities that lessen physical activity and give rise to a sedentary lifestyle, such as television. The present findings also showed that the prevalence of suboptimal physical activity was higher among women than men. It may be mainly a result of beliefs that restrict women’s physical activity mainly to indoor activities.

The findings indicated that 9% of the study population used tobacco, and 3.26% of them smoked tobacco, which is much lower than the prevalence of smoking in the general population of Fars province. This low prevalence of smoking may be result of higher consumption of a smokeless dipping tobacco called *naswar*, as 13% of the target group reported using mouth snuff products.

According to the International Diabetes Federation (IDF), in Middle Eastern and north African countries, prevalence estimates of diabetes range from 3.5% in Yemen to 18.2% in Saudi Arabia [30]. It has been reported around 10% for the Fars province [19]. A meaningfully lower prevalence of elevated FPG (3%) was observed in the study population. This finding is consistent with their higher physical activity and lower rates of obesity, central obesity and dyslipidaemia. However, the present study showed that 5% of them were pre-diabetic. Estimated prevalence of HTN among Afghan refugees (20%) was similar to the reported prevalence at national and provincial levels [19, 31]. This figure indicates that for both FPG and blood pressure status, preventive measures such as lifestyle modifications and medical interventions, if indicated, should be considered.

Laboratory analyses showed that 69% of the refugee population had some degree of abnormality in their lipid profile, i.e. in cholesterol levels, TG levels, or both. According to the Iran STEPS study in 2016, the national prevalence of hypercholesterolaemia and hypertriglyceridemia was found to be 22 and 27.8%, respectively [19]. In a study in Jalalabad in 2013, the prevalence of hyperglycaemia was reported to be 31.4%, i.e., far higher than the prevalence in the Afghan refugee population studied here (13%). This significant difference may reflect the fact that less healthy dietary patterns (e.g., fast foods, junk foods, and high-salt meals) and lifestyle choices (e.g., sedentary workplace, passive hobbies) have not yet become part of refugees’ regular habits.

Two limitations of the study should be noted. First, because of executive and legal issues, this study was conducted in a single refugee camp. According to reports from authorities and experts in this field, social determinants of health in this population – including socioeconomic status, ethnicity, age and gender structure of the population, and access to health – are quite similar to those at most registered settlements located in Iran; however, data security conditions did not allow us to undertake a statistical analysis of these factors. Second, there was no report on national NCD surveillance in Afghanistan, and the prevalence and risk factors for NCDs have been studied so far only in Kabul and Jalalabad City. However, even in these two studies, a representative sample of the whole population was not considered, and the WHO definitions were not used for all risk factors. This issue restricted us to comparing our study population with their peers in the general population of Afghanistan.

## Conclusion

Our study indicated that except for the dietary risk factor of inadequate fruit and vegetable intake, the prevalence of other risk factors was lower than in the Iranian provincial and national population. The study highlights that improving nutritional status deserves more consideration in the refugee population, and that actions are needed to deal with this issue. Fortunately, removing or reducing this risk factor is more feasible than changing lifestyle-related risk factors. Raising awareness about the importance of a healthy diet, along with providing more affordable fruits and vegetables, are two effective steps toward improving dietary status in the refugee population.

## Data Availability

The datasets used and/or analysed during the current study are available from the corresponding author on reasonable request.

## Abbreviations

NCD: Non-communicable diseases
UNHCR: United Nations High Commissioner for Refugees
WHO: World Health Organization
HTN: Hypertension
DM: Diabetes mellitus
HP: Hip circumference
WC: Waist circumference
BP: Blood pressure
LDL-C: Low-density lipoprotein cholesterol
HDL-C: High-density lipoprotein cholesterol
TC: Total cholesterol
TG: Triglycerides
FPG: Fasting plasma glucose
SBP: Systolic blood pressure
DBP: Diastolic blood pressure
IDF: International Diabetes Federation

## References

[1] Matlin SA, Depoux A, Schütte S, Flahault A, Saso L (2018). Migrants’ and refugees’ health: towards an agenda of solutions. Public Health Rev.

[2] Silove D, Steel Z, Bauman A, Chey T, McFarlane A (2007). Trauma, PTSD and the longer-term mental health burden amongst Vietnamese refugees : a comparison with the Australian-born population. Soc Psychiatry Psychiatr Epidemiol.

[3] Vaage AB, Thomsen PH, Silove D, Wentzel-Larsen T, Van Ta T, Hauff E (2010). Long-term mental health of Vietnamese refugees in the aftermath of trauma. Br J Psychiatr.

[4] Giallo R, Riggs E, Lynch C, Vanpraag D, Yelland J, Szwarc J, Duell-Piening P, Tyrell L, Casey S, Brown SJ (2017). The physical and mental health problems of refugee and migrant fathers: findings from an Australian population-based study of children and their families. BMJ Open.

[5] Ackerman LK (1997). Health problems of refugees. J Am Board Fam Pract.

[6] Abbas M, Aloudat T, Bartolomei J, Carballo M, Durieux-Paillard S, Gabus L, Jablonka A, Jackson Y, Kaojaroen K, Koch D (2018). Migrant and refugee populations: a public health and policy perspective on a continuing global crisis. Antimicrob Resist Infect Control.

[7] Rehr M, Shoaib M, Ellithy S, Okour S, Ariti C, Ait-Bouziad I, van den Bosch P, Deprade A, Altarawneh M, Shafei A (2018). Prevalence of non-communicable diseases and access to care among non-Camp Syrian refugees in northern Jordan. Confl Heal.

[8] Agyemang C, van den Born BJ: Non-communicable diseases in migrants: an expert review. Journal of travel medicine 2018. 10.1093/jtm/tay107 30346574

[9] Organization WH: Global status report on noncommunicable diseases 2014. 2014. 10.1161/STROKEAHA.115.008097 25873596

[10] Lopez AD, Murray CC (1998). The global burden of disease, 1990–2020. J Nat Med.

[11] Engelgau MM, Sampson UK, Rabadan-Diehl C, Smith R, Miranda J, Bloomfield GS, Belis D, Narayan KMV, National Health L (2016). Blood institute–UnitedHealth Global Health centers of excellence C: tackling NCD in LMIC: achievements and lessons learned from the NHLBI-UnitedHealth Global Health centers of excellence program. Glob Heart.

[12] De Maeseneer J, Roberts RG, Demarzo M, Heath I, Sewankambo N, Kidd MR, van Weel C, Egilman D, Boelen C, Willems S (2012). Tackling NCDs: a different approach is needed. Lancet (London, England).

[13] Nethan S, Sinha D, Mehrotra R. Non Communicable Disease Risk Factors and their Trends in India. Asian Pac J Cancer Prev. 18(7):2005–10. 10.22034/APJCP.2017.18.7.2005 PMC5648412 28749643

[14] Hoodfar H (2008). The Long Road Home: Adolescent Afghan Refugees in Iran Contemplate ‘Return. J Years Conflict.

[15] Maleki S, Cowan L**:** Iran,Factsheet,1 July 2017. In*.*; 2017: 4.

[16] Abbasi-Shavazi M, Glazebrook D, Mahmoudian H, Jamshidiha G, Sadeghi R: Return to Afghanistan? A study of Afghans living in Tehran, Mashhad, and Zahedan. Retrieved October 30 th 2016. In*.*; 2005.

[17] Tober DM, Taghdisi MH, Jalali M (2006). “Fewer children, better life” or “as many as god wants?” Family planning among low-income Iranian and afghan refugee families in Isfahan, Iran. Med Anthropol Q.

[18] Zahedi A (2007). Transnational marriages, gendered citizenship, and the dilemma of Iranian women married to afghan men. J Iran Stud.

[19] Education IroImoham: Atlas of non-communicable diseases risk-factors surveillance in the Islamic republic of Iran-STEPs 2016,. In*.*; 2016.

[20] Saeed KMI (2014). Prevalence of risk factors for non-communicable diseases in the adult population of urban areas in Kabul City, Afghanistan. Cent Asian J Glob Health.

[21] Saeed KM, Rasooly MH, Alkozai A (2016). Prevalence of risk factors for noncommunicable diseases in Jalalabad city, Afghanistan, evaluated using the WHO STEPwise approach. East Med Health J.

[22] Organization WH: The STEPS instrument and support materials, 2013. In*.*

[23] Riley L, Guthold R, Cowan M, Savin S, Bhatti L, Armstrong T, Bonita R (2016). The World Health Organization STEPwise approach to noncommunicable disease risk-factor surveillance: methods, challenges, and opportunities. Am J Public Health.

[24] WHO: WHO STEPS Surveillance Manual. In*.*; 2017.

[25] Olawuyi AT, Adeoye IA (2018). The prevalence and associated factors of non-communicable disease risk factors among civil servants in Ibadan, Nigeria. PLoS One.

[26] American Diabetes A (2010). Diagnosis and classification of diabetes mellitus. Diab Care.

[27] Jameson JL: Harrison's principles of internal medicine: McGraw-hill education; 2018.

[28] Hadaegh F, Zabetian A, Sarbakhsh P, Khalili D, James WP, Azizi F (2009). Appropriate cutoff values of anthropometric variables to predict cardiovascular outcomes: 7.6 years follow-up in an Iranian population. Int J Obes (2005).

[29] National Cholesterol Education Program (NCEP) Expert Panel on Detection, Evaluation, and Treatment of High Blood Cholesterol in Adults (Adult Treatment Panel III) final report. Circulation. 2002;106(25):3143–421. 12485966

[30] Cho N, Shaw J, Karuranga S, Huang Y, da Rocha FJ, Ohlrogge A, BJDr M, Practice C. IDF Diabetes Atlas: Global estimates of diabetes prevalence for 2017 and projections for 2045. 2018;138:271–81. 10.1016/j.diabres.2018.02.023 29496507

[31] Ahmad Kousha DKE, Jalil Kouhpayezadeh, Kambiz Abachizadeh, Ali Rafei, Fereshteh Salavati, Mahboobeh Darman, Parisa Rezanejad, Abbas Pariani: Iran NCD Risk Factors STEPs Report. In.; 2011.

